# Using vignettes in qualitative research to explore barriers and facilitating factors to the uptake of prevention of mother-to-child transmission services in rural Tanzania: a critical analysis

**DOI:** 10.1186/1471-2288-14-21

**Published:** 2014-02-11

**Authors:** Annabelle Gourlay, Gerry Mshana, Isolde Birdthistle, Grace Bulugu, Basia Zaba, Mark Urassa

**Affiliations:** 1 Faculty of Epidemiology and Population Health, London School of Hygiene & Tropical Medicine, Keppel Street, London WC1E 7HT, UK; 2 National Institute for Medical Research, P.O. Box 1462, Mwanza, Tanzania

**Keywords:** Vignette, Qualitative, Methodology, Africa, Vertical transmission, HIV

## Abstract

**Background:**

Vignettes are short stories about a hypothetical person, traditionally used within research (quantitative or qualitative) on sensitive topics in the developed world. Studies using vignettes in the developing world are emerging, but with no critical examination of their usefulness in such settings. We describe the development and application of vignettes to a qualitative investigation of barriers to uptake of prevention of mother-to-child transmission (PMTCT) HIV services in rural Tanzania in 2012, and critique the successes and challenges of using the technique in this setting.

**Methods:**

Participatory Learning and Action (PLA) group activities (3 male; 3 female groups from Kisesa, north-west Tanzania) were used to develop a vignette representing realistic experiences of an HIV-infected pregnant woman in the community. The vignette was discussed during in-depth interviews with 16 HIV-positive women, 3 partners/relatives, and 5 HIV-negative women who had given birth recently. A critical analysis was applied to assess the development, implementation and usefulness of the vignette.

**Results:**

The majority of in-depth interviewees understood the concept of the vignette and felt the story was realistic, although the story or questions needed repeating in some cases. In-depth interviewers generally applied the vignette as intended, though occasionally were unsure whether to steer the conversation back to the vignette character when participants segued into personal experiences. Interviewees were occasionally confused by questions and responded with what the character *should* do rather than *would* do; also confusing fieldworkers and presenting difficulties for researchers in interpretation. Use of the vignette achieved the main objectives, putting most participants at ease and generating data on barriers to PMTCT service uptake. Participants’ responses to the vignette often reflected their own experience (revealed later in the interviews).

**Conclusions:**

Participatory group research is an effective method for developing vignettes. A vignette was incorporated into qualitative interview discussion guides and used successfully in rural Africa to draw out barriers to PMTCT service use; vignettes may also be valuable in HIV, health service use and drug adherence research in this setting. Application of this technique can prove challenging for fieldworkers, so thorough training should be provided prior to its use.

## Background

Vignettes are short stories about a hypothetical person, presented to participants during qualitative research (e.g. within an interview or group discussion) or quantitative research, to glean information about their own set of beliefs. They are usually developed by drawing from previous research or examples of situations which reflect the local context, creating a story that participants can relate to. Participants are typically asked to comment on how they think the character in the story would feel or act in the given situation, or what they would do themselves. As the focus is on a third person, vignettes can be advantageous in research on sensitive topics where the participant may not feel comfortable discussing their personal situation and may conceal the truth about their own actions or beliefs. They can also, through normalisation of the situation, encourage participants to reveal personal experiences when they feel comfortable to do so [1-4].

Vignettes have traditionally been used in the developed world in (predominantly quantitative) research on psychology and potentially sensitive social and health issues such as sexual health, HIV, mental health, stigmatisation, violence, and in specific vulnerable populations such as children and drug users [1,2,4-13]. Hughes, and Barter and Renolds reflected critically on the methodology of vignettes with reference to their own research and other studies conducted in the developed world, concluding that the technique can be a valuable research tool despite debates surrounding their use: primarily the extent to which vignette responses mirror social reality [1,4]. Studies from the developing world (including Africa) using vignettes have emerged more recently [14-24], but none have critically examined the use of vignettes in such settings. These studies have mainly focussed on similar topics to those investigated traditionally in North America and Europe, such as sexual health, mental health and stigma, but also include areas such as malaria and public health campaigns.

Very little qualitative research about HIV services in this setting, particularly prevention of mother-to-child transmission (PMTCT) of HIV or drug adherence (in the push for universal testing and treatment), has used vignettes to elicit perspectives of patients (or providers) regarding service or drug use. The few examples include Varga and Brookes’ study in South Africa, based on the narrative research method of the World Health Organisation [25]: vignettes were developed during workshops with ‘key informants’ and presented during focus groups and surveys with pregnant HIV-positive adolescents to investigate barriers to participation in PMTCT services [24]. Bentley et al. also used vignettes to investigate perceptions of HIV-positive mothers regarding breastfeeding practices and nutrition in Malawi [26]. Varga and Brookes discussed methodological implications of their approach, reflecting that adolescent mothers spoke more easily about their own experiences after discussing the story of another teenager, and suggesting that in-depth interviews (IDI) exploring personal experiences can be useful in verifying and understanding responses towards the vignette. However, neither paper evaluated the specific challenges nor advantages of applying vignettes in their setting, for example the extent to which respondents understood the directions they were given, or how well fieldworkers facilitated discussions or interviews containing vignettes.

Global commitments have been made to improve uptake of PMTCT services [27] in view of the low coverage noted in many African countries [28]. An emerging body of research is exploring reasons for low access and usage of PMTCT services: barriers include sensitive issues such as stigmatisation regarding HIV status, fear of disclosure to partners or other relatives, and psychological barriers including denial [29].

The potential for reporting bias in studies on barriers to PMTCT service use in sub-Saharan Africa has been noted [29], and in our study setting, under-reporting by women of other socially sensitive outcomes (e.g. number of sexual partners) was reported [30]. We therefore expected that a number of HIV-positive women would not admit to difficulties they faced when accessing PMTCT services, or would feel uncomfortable discussing such issues during interviews. Vignettes could consequently be a valuable and under-used tool in PMTCT/HIV research and drug adherence more widely. They may also offer a contribution to the range of methods available to reduce the social desirability biases encountered with self-reporting of outcomes in HIV, sexual and reproductive health research [31-33]. There is some discussion over whether responses to vignettes may also be socially desirable, particularly when respondents are asked how they themselves would act in the scenario presented. However, asking first how the fictional character would behave and why is thought to reduce the pressure to answer with socially desirable outcomes [4]. In this paper we describe the development and application of a vignette to an investigation of barriers and facilitating factors to uptake of PMTCT services in rural Tanzania. Our objectives for using the method were 1) to create a comfortable environment for IDIs and encourage women to openly discuss difficulties they or acquaintances faced in using PMTCT or maternal and child health services, and 2) to generate data on barriers and facilitating factors to uptake of PMTCT services from the perspective of HIV-positive and HIV-negative mothers, fathers and relatives. We critique the successes and challenges associated with employing vignettes in this setting, in order to determine the feasibility and utility of using this technique in qualitative investigations more widely in sub-Saharan Africa.

## Methods

### Study purpose and context

The study fieldwork was conducted between May and June 2012 in Kisesa, a rural area in north-west Tanzania, to identify barriers and facilitating factors to the uptake of PMTCT services, and ways of overcoming the issues identified. Demographic surveillance and HIV sero-surveillance has been conducted in this community since 1994 [34]. Four health facilities offer antenatal clinic (ANC) and PMTCT services in the community: a health centre in the trading centre (also including an HIV care and treatment centre), and 3 dispensaries in rural villages (providing an intermittent PMTCT service depending on availability of HIV test kits and prophylactic drugs).

### Study procedures

A variety of qualitative methods were used, including participatory learning and action (PLA) group activities, and IDIs incorporating a vignette. Before commencement of the study, fieldworkers received one week of training on relevant research methods and the topic (PMTCT). Training emphasised the participatory element of the PLA activities, as fieldworkers had prior experience of and training in conducting interviews and facilitating focus group discussions, but less experience of leading participatory fieldwork. After familiarisation with the PLA protocol, fieldworkers practised the activities with volunteer participants. The protocol was revised after observing practice sessions and listening to feedback from fieldworkers, (to shorten or simplify some activities), and after conducting the first PLA activity.

### Development of the vignette

The vignette was developed through PLA activities conducted with 3 groups of men and 3 groups of women from different residence areas, each group comprising 8–12 participants. Participants were selected from a sampling frame of men and women aged 15–60 who had at least 1 child. This selection was random, with the exception of a few female HIV-positive individuals (‘seeds’) who were purposively selected from the sampling frame by the principal investigator using the community HIV sero-surveillance data. Female groups included 1–5 HIV-positive ‘seeds’ (see Buzsa et al. for details of the seeded focus group method [35]). Fieldworkers were unaware of the HIV status of all individuals on the recruitment lists and those participating in the activities. Each PLA was facilitated in Kiswahili (commonly spoken national language) by an experienced fieldworker of the same sex as participants. A second fieldworker took notes on the content of discussions, details of the role-play storyline and behaviours of characters, as well as general observations of the group dynamic. The majority of sessions were attended by the principal investigator. Activities were audio-recorded following informed consent from participants.

PLA activities included brainstorming and ranking of barriers, role-playing and group discussion (Table 1). Before the role-plays, fieldworkers facilitated a discussion to identify the central characters that would be involved in a woman’s pregnancy and delivery. Thereafter, the participants were instructed to invent a storyline of a (fictitious) woman who discovers she is HIV-positive at ANC, thinking of the issues that a real woman in their village would face and the decisions she would make when trying to use PMTCT services. Participants then acted the play to the facilitator and observers. De-briefing sessions with fieldworkers were conducted following each PLA activity, informing an initial analysis of emerging themes which was used together with PLA notes by the project investigator to draft the vignette.

**Table 1:** Outline of activities conducted during participatory learning and action group activities

**Section**	**Activity**	**Summary**
1 (day 1)	Group discussion	Discussion focussing on knowledge of vertical transmission of HIV and the PMTCT programme
2 (day 1)	PMTCT ‘journey’	Arrangement of cards representing components of PMTCT services (ANC attendance; HIV testing; provision of antiretroviral drugs to mothers and infants; delivery in the health centre; infant feeding advice)
3 (day 1)	Storyline and role-play	Character and storyline development, role-play of the story composed, followed by group discussion reflecting on the play
4 (day 2)	Barriers brainstorm & ‘wall of challenges’	Group discussion of barriers at each step of the PMTCT ‘journey’ and creation of cards representing each barrier; ranking of barriers by arranging barrier cards on a wall
5 (day 2)	Hanging fruits tree	Brainstorming of possible solutions to overcome the barriers identified, with solutions represented on fruit shaped cards; fruits placed on a tree diagram, according to how easy or difficult to achieve

To compose the vignette storyline, unifying and contrasting elements of the role-plays were identified. Discussions following the role-plays, during which facilitators discussed how realistic the storylines were, were then analysed to confirm unifying elements, or resolve differences between the stories. Themes emerging from other activities, particularly barriers deemed most important in the ranking exercise, were also considered. The final vignette also needed to be viable given the character’s profile, for example, to represent the issues that the character would face considering their residence, marital status or family circumstances. The aim was to present a story that was familiar to most participants (touching on personal experiences, or experiences of acquaintances in their community), but that also achieved the objective of making women feel comfortable to admit to any difficulties they faced (so, for example, a more extreme case of a woman failing to access several of the services was chosen). Overly emotional circumstances or events (e.g. teenage pregnancy or death of a baby) which might derail the interview were avoided.

Once developed, the vignette and associated questions were incorporated into an interview discussion guide, along with open-ended questions about the personal experiences of the respondent during pregnancy, delivery and infant feeding. As conceived in the original study design, fieldworkers then received an additional day of training on the concept and use of the vignette, including examples of other studies employing this technique [24], and on confidentiality (particularly if participants disclosed their HIV status during the interviews). This additional training session was intended to give fieldworkers the chance to familiarise themselves with and discuss the vignette developed from the PLAs, and to ensure the associated methods were fresh in fieldworkers’ minds prior to commencing the interviews. Fieldworkers were asked to review the vignette, and comment on how well it reflected the role-plays and major themes identified from the PLAs (no amendments were suggested). They were instructed to probe for whether responses to the vignette (what participants thought the character in the story would do) reflected real life in their community. After training, fieldworkers practised the questionnaire among themselves and with volunteer participants.

### Use of the vignette

Twenty-one IDIs with HIV-positive (n = 16) and HIV-negative mothers (n = 5) who had recently delivered a child (since 2009) were conducted in Kiswahili by the same fieldworkers that facilitated the PLAs. Mothers were recruited purposively for interview from the PLA activities (and had therefore not necessarily attended clinic-based services, n = 11), and from each of the 4 health facilities in Kisesa by clinic nurses (n = 10). On completion of the PLA activities, each participant was asked to come forward, separately, to receive their travel compensation (5000 Tanzanian shillings, or approximately 3 USD), and asked if they were interested in being contacted for personal interviews in the future. Facilitators only scheduled specific appointments for interview with selected HIV-positive and negative participants, based on coded lists prepared by the principal investigator using community surveillance data. Facilitators were unaware of the HIV status of participants at the time of recruitment. For the clinic-based recruitment, each nurse was asked to invite and schedule interview appointments for at least two HIV-positive women who were pregnant or had recently given birth, during private consultations with their clients at antenatal or child follow-up clinics. Researchers did not have access to any clinic data for the recruitment.

Three interviews with partners/relatives of HIV-infected mothers were also conducted: women who had disclosed their HIV status during the IDIs were asked if their male partner, or otherwise a female relative, could be contacted for interview.

The same vignette was used in all interviews, and was read out to participants. Interviews lasted between one and three hours, and were audio-recorded after obtaining consent from the participant.

### Critical analysis

Critical analysis of the vignette methodology was guided by the following key questions:

1. Was the vignette method developed and implemented as intended? This includes how well the vignette was developed for the study context, delivered by interviewers and received by participants, in order to assess the feasibility of the approach. To answer this evaluation question, we: (a) reflected on the successes and challenges in developing the vignette; and (b) assessed IDI transcripts for any difficulties in interpretation of the vignette by the participants or fieldworkers, including confusion, misunderstandings or delays during the vignette section of the interviews, and whether participants considered the final vignette to be realistic. In analysis of the transcripts (audio-recordings were transcribed verbatim, translated into English, and the resulting data managed using NVIVO 9), codes were created to capture the way participants responded to the vignettes, and how fieldworkers dealt with their answers.

2. Did the vignette method achieve its intended objectives? To this end, we gauged from transcripts whether the vignette helped to: (a) make participants comfortable during the interview, e.g. to discuss their personal experiences with ANC/ PMTCT services and HIV status; and (b) generate useful findings (data) about barriers and facilitating factors to PMTCT uptake, analysed through a framework approach which included thematic analysis to develop the coding scheme for barriers to PMTCT service uptake. We considered data quantity and quality, including any difficulties in interpretation of the data during analysis.

### Ethical approval

This study was approved by the Lake Zone ethical review board of Tanzania, the Tanzanian Medical Research Coordinating Committee, and by the London School of Hygiene and Tropical Medicine ethics committee.

## Results

### Summary of the vignette developed

The final vignette described the story of a pregnant woman living in a remote rural village who discovers her HIV-positive status at ANC, faces negative reactions from her partner upon disclosure of her HIV status, is unable to return to the clinic for further PMTCT services (including anti-retroviral drugs) and gives birth at home fearing involuntary disclosure to other relatives. The story was split into 3 sections, with questions after each section about what the woman would most likely do in her situation. Details of the vignette and questions used in the in-depth interviews, excluding probes, are presented in the following section.

#### Details of the vignette

I’d now like to tell you a story about a pregnant woman called [Flora] and her experiences in trying to access antenatal clinic (ANC), delivery and infant health services. I will tell you part of the story, then I would like you to help me complete the story.

Flora lives in a remote village in Welamasonga, she is 27 years old. She is married to Paulo and she has 3 children. She becomes pregnant and after a few months decides to attend an antenatal clinic by herself. At the ANC she receives a test for HIV. The nurse tells her that she is HIV-positive but explains that there are medicines that she can take to save the baby from being infected with HIV. She also tells Flora that it is important that she delivers the baby in the health centre so that it can also receive medicine to reduce the chances of it being infected. She gives the woman the medicines to take during her pregnancy, and also tells her to persuade her husband to come for an HIV test. She also discusses options for feeding the infant, and advises Flora to breastfeed the child for 6 months without any replacement food. The nurse explained all this information very quickly.

What do you think happens next? Please think for Flora, as a woman in your community, and imagine what she would be thinking and feeling at this time.

In the next part of the story, Flora goes home to her husband and tells him the result of her HIV test, and what the nurse advised her. He is angry and denies her status because he believes he is not infected, and questions whether she has had other partners. Flora decided to disclose her status to her sister and get her support, but she decides not tell to any of her other relatives about what happened.

Do you think Flora would go back to the clinic for more ANC appointments? Why/why not?

Do you think Flora would be able to go to the HIV care and treatment clinic? Why/why not?

Do you think Flora would be able to take the treatments during her pregnancy? Why/why not?

Where do you think Flora will give birth to her child? Why?

Do you think she would be able to swallow the HIV medicines during labour and delivery? Why/why not?

I’ll now tell you the next part of the story:

Unfortunately Flora didn’t manage to take the medicines during her pregnancy because she feared the reaction of her husband. She gave birth at home because she was unable to get the support of her husband for the transport fare and to buy gloves and other items which might be needed when she arrives at the delivery ward. She also fears the suspicion of her relatives who might escort her to the delivery ward: they might see her swallowing the HIV medication during labour pain, and she might have to wash her own clothes or sheets after delivery.

Do you think Flora will be able to take the baby back to the clinic for ARVs in the first few days after it is born? Why/why not?

Will she be able to take the baby to a clinic to be tested for HIV after one month? Why/why not?

Will she be able to follow the advice about breastfeeding? Why/why not?

Does Flora’s story reflect what can happen in real life? Why/why not?

### 1. Was the vignette method developed and implemented as intended?

#### 1a. Lessons from developing the vignette

The original protocol for the PLA storyline and role-play activity started with participants developing a tree diagram, where participants discussed all possible outcomes at each step of the PMTCT service chain and agreed on the most likely scenario (using the approach of Varga et al. [24]). However, fieldworkers and participants struggled with this approach during practice and piloting, and the activity exceeded the allocated time. The activity was therefore simplified: participants were given a starting point (a pregnant woman discovering her HIV-positive status at ANC) and ending point (delivery of the child, and potentially accessing infant PMTCT services after delivery), and encouraged to create their own story. The majority of groups easily grasped the new instructions for creating and enacting the storyline, while a few groups required further guidance from the facilitator initially.

The participatory group work was instrumental in developing a vignette that was locally relevant. The role-plays generated content for the vignette, and the importance of certain issues came to light through the observation of characters’ behaviour. However, it could be argued that other PLA activities and discussions, aside from the role-plays, were equally useful in developing content for the vignette and helping to ‘merge’ multiple role-plays into one final story – a process which presented challenges. For example, as expected, there were differences between the storylines from each group. Discussions following the role-plays occasionally revealed that elements of the storylines did not reflect real life, and were therefore important in resolving differences between the plays. Deciding whether or not to include themes/scenarios that emerged in only one or two plays was also challenging. Other activities such as barriers brainstorming and ranking exercises were valuable in these decisions: themes that emerged infrequently in the plays, but that were ranked as highly important by several groups, were selected for inclusion in the final vignette.

#### 1b. Lessons from implementing the vignette – was it delivered and received as intended?

The majority of in-depth interviewees understood the concept of the vignette and follow-up questions, although some had difficulties understanding and the story or questions had to be repeated. Some participants (a minority) said they had ‘failed’ to understand or give an answer, remained silent, or asked the interviewer to help them when asked what Flora would do or be thinking. One respondent who had difficulties understanding the vignette arrived late for the interview and appeared tired before beginning, while another had limited knowledge of Kiswahili, based on de-briefing discussions. However, in most cases interviewers re-phrased the story or instructions and the respondent grasped the concept.

*I: …She [Flora] has now returned home, what in general do you think will happen afterwards?R: Maybe a quarrel with her husband - Her husband refusing to go to test. (HIV-negative mother)*

*I: …Yes, this is a story about Flora… What do you think happened afterwards?R: [silence]*

I: Have you understood the story well or shall I repeat it so that you may understand it?

R: Yes, please repeat it. (Male partner)

*I: …I want you to tell me if there is anything which will prevent Flora [from going to the clinic]…R: Mm, I have failed to give the answer.*

I: Don’t you know what it is called in Kiswahili? (HIV-negative mother)

Further engagement with the story was illustrated by one respondent who referred to Flora spontaneously later in the interview (after the vignette discussion): “*Like we said about Flora, she went there [the clinic] alone”.* Interviewers also referred to Flora within the context of discussions about personal experiences and perceptions of PMTCT/antenatal service provision.

Interviewees occasionally relayed what the character *should* or *must* do, offering her advice, rather than what they thought she *would* actually do. Further questioning sometimes clarified perspectives, but on other occasions yielded similar responses.

*I: Is there perhaps anything that has made Flora fail to swallow the drugs?R: No there isn’t. Perhaps she should just continue taking them, she shouldn’t stop taking them. (HIV-positive mother)*

*R: According to my opinions…the only way is to use medicine.I: Yes, you are saying that according to your opinions…Now we want your opinions but you have to involve Flora…R: I would only advise Flora to continue using… [the drugs]. (HIV-positive mother)*

The quote above also illustrates a challenge faced by the interviewers - how to steer the conversation when respondents spoke of their own beliefs, or actions, rather than what Flora would think or do. In some cases, the interviewer cut off replies expressed in the first person and immediately returned the conversation to Flora’s perspective.

*R: I would not tell anybody [test result].I: No, it is Flora, not you. We are first talking about Flora. (HIV-negative mother)*

However, fieldworkers mostly probed further into personal experiences before returning to the vignette.

Four participants did not think the vignette overall was realistic, and a few others did not think Flora would face many challenges with participating in the PMTCT programme despite her circumstances. These were typically HIV-positive women who had reported accessing PMTCT services and complying with appointments themselves, as well as a few HIV-negative women and both male partners.

*I: And do you think the story represents actual life?R: No it does not represent itI: Why?R: Because Flora…she stopped going to use the medicine. (HIV-positive mother, used ARVs during pregnancy)*

However, most participants agreed that the vignette was a realistic example of the issues faced by an HIV-infected woman in their community. Participants sometimes anticipated the next section of the vignette. For example, several women predicted that Flora’s husband would react badly to her HIV results, including refusing to test or blaming her, while a few anticipated that Flora would deliver at home.

*I: Do you think it [the story] shows the actual life of many women who are pregnant… and discovered to be HIV-positive?R: This story shows the truth. (HIV-positive mother)*

Responses to the vignette were compared across interviews, between HIV-positive and negative women, and between mothers and male partners/relatives. However, there were few identifiable differences in reactions to the vignettes by respondent type or place of recruitment.

### 2. Did the vignette method achieve its research objectives?

#### 2a. Did the vignette make participants more comfortable during IDIs?

For the IDI discussion guide with mothers, the vignette was placed after personal background questions (place of residence, marital status and children), but before the section on personal experiences with ANC services. It was hoped that participants would talk more freely about their own experiences and admit to difficulties that they or acquaintances faced in using PMTCT services, after hearing the story and challenges of another woman in their community. For the partner/relatives IDI discussion guide, the vignette appeared *after* the section on personal experiences (of assisting their female partner/relative during their pregnancy), because the descriptions of negative partner reactions and lack of support might influence partners’ responses (e.g. over-stating their involvement).

Five out of sixteen known HIV-positive women voluntarily disclosed their status to the interviewer before or during the vignette discussion, and gave examples of their own experiences receiving HIV-positive test results or using PMTCT services. Another seven voluntarily disclosed their status later in the interview, while discussing their own experiences of antenatal care. Three said they had tested HIV-negative, and one said she had not received an HIV test at ANC. Several participants (HIV-positive and HIV-negative) also described experiences of HIV-positive relatives or friends.

*I: Can there perhaps be an obstacle that can make her fail to deliver at the hospital?R: YesI: Like what obstacle?R: For example myself, I delivered at home because it was at night. The birth pains started at night and there was no one to take me to the hospital… (HIV-positive mother)*

*I: And do you think that what we have been talking about together is what occurs in our community?R: I have happened to see one – there is one woman. She was born having HIV…They started giving her medicine but she failed to swallow the tablets and instead she was throwing them away. (HIV-negative mother)*

A few participants appeared to be unsettled or uncomfortable during the vignette discussion. For example, some respondents said they had ‘failed’ (as illustrated above, Section 1b). One respondent (known HIV-positive but who did not disclose her status) seemed unwilling to answer some probing questions that verified if the response was realistic, although she appeared willing to answer subsequent questions.

#### 2b. Did the vignette generate data about barriers to PMTCT service uptake?

Discussions during the vignette sections of all IDIs produced data that could be coded to inform the analysis of barriers and facilitating factors to PMTCT service uptake [36]. Most data came directly from probes asking what challenges or motivating factors Flora would face at each step (e.g. returning to the clinic or taking medicine).

*I: ....What challenges do you think Flora will encounter?R: There will be challenges at the time when she is going to deliver. Sometimes the health centre may be far away from home..... (HIV-negative mother)*

Data was also generated indirectly, for example when asking ‘what would happen next’, after reading the first part of the story, or probing for what Flora would be thinking or feeling.

*I: What do you think this woman will be thinking of.....?R: She will just be thinking - Because she has already told her elder sister [her result], her elder sister will be giving her advice just to use those drugs so that you protect the baby. (HIV-positive mother)*

The vignette provided context and situated the discussions. This allowed respondents to share their own experiences, or those of others, including barriers or facilitating factors. It also facilitated discussion of barriers to PMTCT service use among HIV-positive women who did not disclose their status to the interviewer, and among HIV-negative women based on their experiences of pregnancy and maternal and child health services. The perceived reality of the vignette (illustrated in Section 1b) also affected data generation: where respondents thought the vignette was realistic, they sometimes gave reasons that could be coded as data.

*I: Why did you say that this story really shows the things that occur?R: This story shows the truth, and it usually occurs in the family…there are others…she can go with her husband for treatment. There is another [partner] who can refuse and then a quarrel ensues. (HIV-negative mother)*

Comparison within cases between the vignette and personal experiences sections of the IDI revealed that responses to questions about the vignette often reflected the respondent’s own experiences. For example, one female participant told her husband about her positive HIV test result, but he refused to test and deserted her. After hearing the first part of Flora’s story and being asked what would happen next, she replied: *“She [Flora] can tell him [her husband]… You know, some men if you tell them, they become angry…Some do not want to show up at the service.”* Another participant was asked if Flora could disclose her test results, and responded: *“No…She will decide to remain quiet…she wants to see first…if it is true”*. This participant later revealed that she had initially denied her own positive HIV test result and delayed disclosure to her partner.

When respondents answered with what they thought Flora *should* do (Section 1b), this also presented difficulties in interpretation of the data for the researchers (for example, if the participant suggested reasons why she should access services (potential facilitating factors), it was not clear if these were realistic).

## Discussion

This is, to our knowledge, the first methodological paper to critically examine the development and use of vignettes in Africa, and one of few studies to apply this technique within the context of HIV research in Africa. Overall, the development of vignettes through participatory group work, and use of vignettes within IDIs by locally trained fieldworkers, was feasible and useful in this setting. We believe it could be a valuable tool for future qualitative research in the field of PMTCT and other health or social issues in Africa.

Storyline development and role-play was a practical way of generating ideas for the vignette, although a simplified, more structured approach was required. It is possible that the more open-ended approach used by Varga et al. in South Africa was feasible in their study because participants had a greater knowledge of PMTCT services and a higher level of education: respondents included health workers, and eligibility was based on having “experience and knowledge of the health issue”, while our study included rural community members with no experience of the programme. Alternatively, differences in the experience level of fieldworkers may explain the variation in success of this approach. While our fieldworkers were generally experienced in qualitative methods, including focus group discussions and IDIs, they had less experience of participatory methods and no experience of using vignettes. In addition, they had been involved in HIV research, but were less familiar with PMTCT specifically. Intensive training was provided, but more practical experience, including more time for practice and piloting prior to the fieldwork, may have been needed to better facilitate the storyline development and role-plays.

While our approach to creating the storylines was simpler for the facilitators and participants, decisions of what to include in the final vignette were not straightforward. However, triangulation with results from other activities and discussions during the PLAs facilitated this ‘merging’ process. It could also be reasoned that the final vignette does not need to represent the majority of women in the community, but should at least be a realistic example of some women, so that it can successfully be used to build discussion in the interviews and encourage women to admit to their own experiences.

Most interviewees appeared to understand the concept of the vignette. This may partially reflect the fact that roughly half of the interviewees had participated in the PLA activities used to generate the vignette, although a similar proportion of interviewees recruited by other means comprehended the task. Prior participation in the PLAs may also have affected responses to the vignette more generally, for example coloured by views of other PLA participants regarding PMTCT service use, though interviewees would only have recognised small elements of their role-play in the vignette. Believing the vignette was realistic or anticipating the next section of the vignette may also reflect comprehension of the story, while this also generally facilitated the discussions.

The minority of cases where the story had to be repeated, or instructions had to be clarified, may have been due primarily to unclear questioning by interviewers, language barriers (poor command of Kiswahili), general shyness, or lack of readiness for the interview, rather than difficulties with the vignette technique itself. Encouraging the participant to think of the character as a woman in their community was especially helpful in enabling them to grasp the concept. Allowing time for the participant to digest information in the vignette and to seek clarification before proceeding with any questions or discussions may also be beneficial, particularly with longer vignettes [1]. A few participants misinterpreted questions about the vignette, thinking they were being asked what Flora *should* do, while fieldworkers also found these unexpected responses confusing (discussed further with regard to data interpretation below).

Use of the vignette achieved the main objectives. Firstly, we hoped that the vignette would encourage participants, particularly HIV-positive women, to feel comfortable and freely discuss their own situation and any difficulties they faced in accessing maternal health or PMTCT services: several respondents offered examples from their own experience, or that of family or friends, or commented on what they would do in Flora’s situation. Renolds noted that respondents were encouraged to voice more extreme concerns when the story was real [2]. While our vignette did not give a biographical account of one person, it was based on discussions with the community and their own stories, and was considered realistic by the majority of interviewees. This emphasises the utility of developing the vignette through participation of community members. While the expression of personal experiences was a benefit, interviewers occasionally struggled to deal with this and were reluctant to digress from the discussion guide and Flora’s perspective. Fieldworker training should therefore stress the importance of drawing out the respondent’s own experiences, before returning the conversation back to Flora. Ideally, transcripts should be analysed during the course of fieldwork to identify and deal with these issues immediately. The use of questions such as “does this really happen in your community?” was particularly effective in drawing out personal experiences.

An unusual and interesting feature of this study was the knowledge of participant HIV-status by the principal investigator, thus allowing exploration of whether the technique may have encouraged or hindered disclosure of positive results. Most women disclosed their positive status during the vignette or personal experiences section of the interview, although they may have disclosed their status regardless of whether or not the vignette was included. A few women disclosed their status to the interviewer before the vignette discussion, in which case the vignette may not have offered any extra benefit. Some participants chose not to disclose their status (or they had not received their results, or research testing results were false positives). Therefore it is possible that presenting the case of a woman diagnosed with HIV who faces difficulties accessing PMTCT care, is sometimes insufficient to encourage disclosure. However, in such situations, the vignette at least enables discussion of the topic in a non-threatening way in the third person. While other factors will influence disclosure during the interview (such as the interviewer, the environment in which the interview is conducted, the respondent’s disclosure history and their willingness to disclose to strangers), the majority of HIV-positive respondents disclosed their status during or following the vignette discussion, suggesting that the vignette may have contributed to creating a comfortable atmosphere for the interviewee.

It is worth noting that a few participants said they had ‘failed’ to answer questions, suggesting that they perhaps felt ‘tested’ by the questions. This has been described in vignette studies from North America and Europe, particularly when respondents felt the story outcomes differed from what they had anticipated [1]. In order to avoid making participants feel nervous, it may therefore be important to reiterate that there are no ‘wrong’ answers in the introduction: an approach adopted in one study in Ghana [16].

Secondly, the vignette facilitated the discussion of barriers to using PMTCT services by focussing on a third person: most participants spoke freely about potential challenges that Flora would face. This meant that barriers could be discussed in all the interviews, including those with HIV-negative mothers, partners and relatives who had no direct experience of PMTCT services, but who were able to contribute useful information based on experiences of acquaintances, or their own experiences of maternal and child health services (into which PMTCT services are usually integrated). This advantage has been noted previously in developed world studies [2], and also contributed to boosting the quantity of data generated.

The direct comparison between the vignette and personal experiences section of the IDIs was another strength of this study, contributing to an understanding of the extent to which responses to the vignette (what Flora would do) reflected participants’ own actions and thus data quality. Participants’ responses to the vignette often appeared to mirror their own experiences. This suggests that the vignette can be a useful tool to capture (indirectly) the perceptions and actions of shy respondents, for example HIV-positive individuals who do not wish to reveal their status or personal experiences. It also suggests that vignettes may be a valuable method for reducing the social desirability biases associated with self-reporting in HIV and reproductive health research.

Some participants gave advice to Flora and stated what she *should* or *must* do, and it was only through further questioning that what she *would* do, or likely difficulties that she would face, came to light, if at all. This may reflect the respondent’s own actions, or illustrate a social desirability bias (what they think they should have done themselves). This distinction between ‘belief’ and ‘action’ is important and is one of the most common problems reported in developed world studies when using vignettes and interpreting their findings [1,2]. However, Renolds and Finch argue that the process of the discussion is more important than the stated outcome/action, and that vignettes can still yield useful information, particularly when integrated with other methods such as interviews [2,3]. None-the-less, interviewers should be prepared for and probe further when ‘*should’* responses are given, to determine if the answer is realistic. While this was included in training for our fieldworkers, further emphasis and practice may be required.

## Conclusions

Participatory group research is an effective method for developing vignettes. Vignettes can be incorporated into qualitative interview discussion guides and used successfully in rural African settings to draw out barriers to PMTCT service use, indicating potential usefulness in other areas of research on HIV, health service use, and drug adherence. This method is often overlooked in HIV research, and should be considered more often. Issues experienced with the technique largely mirror those reported in developed world settings. Fieldworkers experienced in qualitative research methods but without prior experience of vignettes can be used. However, application of this technique can prove challenging so supervision and thorough training should be provided, including the importance of probing for the reality of the suggested outcome, and preparation for the different ways that participants may respond to the vignette questions, particularly when personal experiences are brought up. Methods (e.g. participatory group work) to develop vignettes must also be carefully piloted.

## Competing interests

The authors declare that they have no competing interests.

## Authors’ contributions

AG designed the study, managed the fieldwork, analysed the data and wrote the manuscript. AG and GM conceived the idea for this paper. GM gave advice on fieldwork materials and procedures, and assisted with fieldworker training and de-briefings. IB advised on fieldwork materials and gave extensive feedback on early manuscript drafts. IB and GM helped interpret the findings. GB facilitated female PLA activities and interviews, and helped with recruitment. MU (Kisesa cohort study director) facilitated coordination of daily activities. MU and BZ (senior technical advisor for Kisesa cohort activities) provided overall guidance. All authors reviewed and agreed to the final version of this manuscript.

## Pre-publication history

The pre-publication history for this paper can be accessed here:

http://www.biomedcentral.com/1471-2288/14/21/prepub
